# Psychological experience of health care workers in an anaesthesia and intensive care units

**DOI:** 10.1192/j.eurpsy.2026.10795

**Published:** 2026-07-31

**Authors:** N. Chaouch, W. Gdich, S. Bey, G. Amri, H. Ziedi, R. Ghachem, N. Mechergui

**Affiliations:** 1 Occupational Medecine, Habib Thameur Hospital, Tunis; 2 B Department, Razi Hospital, Manouba, Tunisia

## Abstract

**Introduction:**

Health care workers (HCWs) in anaesthesia and intensive care units are constantly confronted with unexpected, complex and sometimes intense emotional situations.

**Objectives:**

To assess the level of perceived stress among HCWs in an anaesthesia and intensive care unit and to determine the prevalence and intensity of anxiety-depressive symptoms in this population.

**Methods:**

Cross-sectional study conducted among HCWs in the Anaesthesia-Intensive Care Unit of the Habib Thameur Hospital in Tunis during July 2024, using the Perceived Stress Scale (PSS-10) to assess stress levels. Screening and assessment of the severity of anxiety-depressive symptoms were carried out using psychometric scales, the General Anxiety Disorder-7 (GAD-7) and the Patient Health Questionnaire-9 (PHQ-9).

**Results:**

Our population was predominantly female (75%) with a mean age of 35 +- 8 years. They were mainly paramedical staff (72.5%). The median professional seniority was 8 years. Working hours were atypical in 50% of the cases. Stress levels were moderate in 65% of cases, severe in 32.5% and low in 2.5%. Anxiety symptoms were present in 85% of HCWs, with mild to moderate anxiety in 65% of cases. Depressive disorders were present in 89% of HCWs. It was moderate to severe in 61.5% of cases. Moderate depression was associated with having an outside job (p=0.035) and with educational level (p=0.031). There was a non-statistically significant correlation between high levels of stress and severe depression (p=0.05)

**Conclusions:**

Our study showed that the level of perceived stress, frequency and severity of anxiety-depressive symptoms were high among HCWs in the anaesthesia and intensive care unit. Early detection of anxiety-depressive disorders and implementation of preventive strategies are essential to ensure better mental health among HCWs

**Disclosure of Interest:**

None Declared

